# Who Bears the Burden? Exploring Demographic Disparities in Drug-Related Nuisance Property Ordinances Across the United States

**DOI:** 10.21203/rs.3.rs-10472941/v1

**Published:** 2026-08-05

**Authors:** Pooja Shah, Jaskiran Dhinsa, Katherine Wheeler-Martin, Corey S. Davis, Ashleigh Dennis, Moumita Chowdhury, Charles DiMaggio, Magdalena Cerdá, Spruha Joshi

**Affiliations:** NYU Grossman School of Medicine: New York University School of Medicine; NYU Grossman School of Medicine: New York University School of Medicine; NYU Grossman School of Medicine: New York University School of Medicine; Network for Public Health Law; Network for Public Health Law; NYU Grossman School of Medicine: New York University School of Medicine; NYU Grossman School of Medicine: New York University School of Medicine; NYU Grossman School of Medicine: New York University School of Medicine; University of Michigan School of Public Health

**Keywords:** Nuisance Law, Eviction, Demographic Disparities, Local Drug Law, Legal Epidemiology

## Abstract

Nuisance property ordinances are municipal laws that penalize property owners or renters for alleged criminal activity or repeated disturbances, including emergency calls. As these laws have expanded, concerns have grown that they may disproportionately impact marginalized populations, including people who use drugs, by targeting drug-related activities and exacerbating chronic housing instability. This study examines the demographic and municipal-level characteristics associated with the presence of drug-related nuisance property ordinances (DNPOs) across major U.S. municipalities. Municipal codes were systemically analyzed for DNPOs across 482 municipalities in the U.S. DNPOs were classified as ordinances that list specific activities related to drugs, drug paraphernalia, or illegal activity as a nuisance. A chi-square test was used to compare proportions of demographic and socioeconomic characteristics between municipalities with and without DNPOs. 202 of 482 municipalities had DNPOs (41.9%). 73 of 202 municipalities, 36.1% of DNPOs, authorized eviction as a legal method of abatement. DNPOs were more likely to be found in municipalities with higher proportion of Black, non-Hispanic populations, lower median household income, higher poverty rates, higher unemployment rates, higher population density, higher proportions of renters, and moderate to higher rates of overdose (p < 0.05). DNPOs are prevalent across U.S. municipalities and frequently include eviction as a permitted or required abatement strategy. Our findings show that DNPOs are more likely to be present in socioeconomically disadvantaged communities. These findings underscore the need to further evaluate the health and equity consequences of nuisance property ordinances for people who use drugs.

## 1. INTRODUCTION

Nuisance property ordinances are municipal laws that penalize people for reports of disruptive or criminal activity or repeated emergency calls on residential properties^1,2^. Nuisance property ordinances, which have burgeoned in number and scope since the late 1980s, enumerate “nuisance activities” and designate properties, from which one or more such activities originate, as nuisances^1,3,4^. Typically, once a property has been designated as a nuisance, one or more responsible parties are required to abate the nuisance or face penalties^3^. Although specific provisions vary across municipalities, landlords are commonly held as one of these responsible parties and are strongly incentivized by ordinances to formally or informally evict tenants to avoid penalties such as fines, property forfeiture, or imprisonment^2,3^. In some cases, nuisance property ordinances explicitly require eviction as a means of abatement^3^.

As renter-occupied housing has increased over the past two decades^5,6^, the potential reach and consequences of nuisance property ordinances have become increasingly salient. The use of eviction as an abatement pathway places tenants at risk of housing insecurity^7^, a condition associated with adverse health outcomes and elevated mortality; across over 500 U.S counties all-cause mortality increased 9.32 deaths per 100,000 for every 1% in eviction^8^. These outcomes are further exacerbated among marginalized populations^9–11^ particularly those already vulnerable to frequent and consequential interactions with emergency services, law enforcement, and housing systems^3,7,12,13^. Nuisance property ordinances that attach penalties to repeated service calls or perceived criminal activity at a residence may disproportionately affect individuals engaged in stigmatized or criminalized behaviors, such as drug use^3,7,12,13^. Although much of the existing research on nuisance property ordinance implementation and impact has focused on domestic violence survivors and individuals with mental health conditions^1,2,14^, people who use drugs (PWUD) are, thus, also a crucial population of interest in regard to the impact of nuisance property ordinances^13^.

While previous research has examined the implementation and consequences of nuisance property ordinances, this literature has examined the sheer presence of nuisance ordinances largely. Nuisance ordinances, however, encompass a wide range of specific activities, and relatively little attention has been paid to the nationwide presence of ordinances specific to drug-related behaviors. Emerging evidence suggests that drug-related activity may represent a key pathway through which nuisance property ordinances are enforced. Qualitative research from Vancouver’s Downtown Eastside, for example, found that tenants with histories of drug use were routinely surveilled and evicted for conduct unlikely to prompt enforcement against other residents^15^. In many municipalities, nuisance property ordinances were found to encourage or require landlords to evict tenants for alleged or perceived drug activity, even in the absence of criminal charges^11,15^.

This distinction is particularly important given the extent to which nuisance designations are often initiated by calls for emergency services, including those related to overdose and other drug-related events. A legal review of criminal activity nuisance ordinances across Ohio found that 10–40% were precipitated by overdose-related calls, which can serve as grounds for eviction^7^. In one case, a woman who contacted law enforcement twice for overdose intervention received notice that her property had been designated a nuisance; eviction proceedings began the same day^13^.

The intersection between nuisance ordinances and PWUD is particularly important given the rates of drug use and overdose in the United States. In 2023, 16.8% of individuals aged 12 and older reported past-month use of substances classified as Schedule I^16^, a 1.9% increase from the previous year. Despite recent declines, drug overdose rates remain high, with just over 70,000 predicted year-over deaths as of November 2025^17^. Currently, the link between housing instability and drug-use and drug-related mortality is highly pronounced, with overdose classified as the leading cause of death among people experiencing homelessness in 2024; some studies cite 45% of deaths among on the unhoused population as attributed to fatal overdose^18,19^. Within this context, the eviction pathway authorized by nuisance property ordinances could create conditions in which drug-related activity triggers chronic housing instability and worsens already poor health outcomes^20,21^ Because these ordinances frequently rely on calls for service as triggers for enforcement, overdose events and other drug-related emergencies may disproportionately initiate nuisance designations, effectively penalizing calls for help in an overdose.

Moreover, a small body of research has documented how the implementation and enforcement of the same nuisance property ordinances differ between communities. This work demonstrates pronounced racial variation in nuisance law implementation and enforcement^3,13^. In Milwaukee, for example, properties in predominantly Black neighborhoods were significantly more likely to receive nuisance citations than those in predominantly White neighborhoods; approximately 1 in 16 eligible properties in Black neighborhoods received a citation compared with 1 in 41 in non-Hispanic White neighborhoods^3^. When discussing adoption of a nuisance property ordinance in Bedford, Ohio, which had recently experienced a large racial demographic shift, lawmakers explicitly referenced these changing racial demographics as a motivating factor in the decision to enact policy^22^.

Research concerning the prevalence, scope, and distribution of nuisance property ordinances across the United States remains limited. The first national legal analysis of nuisance property ordinances, which surveyed the 40 largest U.S. cities, found that 36 had enacted such ordinances^2^. Beyond this, however, little is known about how drug-related nuisance provisions are distributed across municipalities or the community characteristics associated with their adoption. This study addresses these gaps through a descriptive analysis of community characteristics associated with the presence of drug nuisance property ordinances amongst the largest cities across the U.S, contributing to understanding of their distribution and potential consequences.

## 2. METHODS

A dataset was created of local nuisance ordinances across the largest U.S. cities by population. All cities with populations > 75,000 based on 2016–2020 5-year American Communities Survey (ACS) estimates were included, resulting in a total of 471 cities^23^. Additionally, to ensure representation from all 50 states, 10 cities with populations < 75,000 and five census-designated places (e.g., Honolulu, HI) were included for states that do not contain cities with populations > 75,000, yielding a total of 486 municipalities. Relevant local laws could not be accessed for four municipalities in the sample, resulting in a final sample size of 482 municipalities. The population of municipalities in the dataset totaled 109.3 million, 32.8% of the United States population as of 2020.

### 2.1 Data Collection

A data collection instrument was created to systematically collect municipal nuisance ordinances related to drug use and code the activities triggering a nuisance designation and the penalties imposed for violation. The instruments and search protocol are available through written requests.

Public health attorney co-authors devised a list of activities and terms that could either directly or indirectly penalize drug activity on a residential property prior to the commencement of data collection. For all 482 municipalities, coders systematically searched publicly available code repositories for local ordinances that deem drug activity, paraphernalia activities specifically, and/or illegal activity generally to be a nuisance as of December 31, 2022. Such laws are referred to as a drug-related nuisance property ordinance (DNPO). Coders also noted where these ordinances deemed disorderly conduct, disturbing the peace, and non-drug related loitering to be a nuisance, as these terms could function as a proxy for drug-related behavior. Where applicable, enumeration of specific drug-related activities (simple possession, use, sale, distribution, delivery, manufacturing, advertising, cultivation, loitering, “other”) were also coded. Simple possession and use for drug or drug paraphernalia activity were identified as the most relevant sub-activities for descriptive analysis as they represent activities for personal use of drugs and highlight ordinances not focusing on persons selling or manufacturing drugs on residential properties.

### 2.2 Drug-related Nuisance Property Ordinances

Each municipality was characterized based on the presence or absence of a DNPO, whether the ordinance authorized eviction as a method of nuisance abatement, and whether violations of the ordinance could result in criminal penalties, non-criminal penalties, or both. Criminal penalties were selected if any of the following penalties were explicitly enumerated in the ordinance as a violation for committing a nuisance activity: misdemeanor, incarceration, criminal fines, or offenses. Non-criminal penalties were all penalties enumerated that did not fall into these categories.

### 2.3 Municipal characteristics

Community characteristics included municipal-level economic indicators, U.S geographic subregions, population demographics, arrests rates, and overdose mortality. Economic and population demographic data were obtained from the ACS 5-year estimates 2016–2020 for all 482 cities^23^. Economic indicators included were median household income, poverty level, Gini index, and unemployment rate. Poverty rates and unemployment rates were calculated as rates of the total population within each municipality. Population demographics included race, population density, and percentage renters. Due to small cell sizes, several racial categories (American Indian or Alaska Native, Asian, Native Hawaiian or Other Pacific Islander, and “Some Other Race”) were combined into a single category (“Other race, non-Hispanic”). Percent renters were calculated as the proportion of renter-occupied relative to owneroccupied housing units within each municipality. Subregions were determined from the U.S census^24^.

Municipal level arrest data were obtained from the 2023 National Incident-Based Reporting System (NIBRS)^25^. Data was available for 425 of the 482 municipalities in the dataset. Arrests were classified into three mutually exclusive categories: drug-related offenses, property crime offenses (excluding fraud and other financial crimes), and public order offenses. Drug-related offenses were defined as NIBRS drug/narcotic offenses, including drug/narcotic violations and drug equipment violations. Public order offenses are also defined by NIBRS and include prostitution-related offenses, curfew/loitering/vagrancy violations, disorderly conduct, driving under the influence, and liquor law violations^25^. Arrest rates were calculated per 100,000 populations.

Three-year average annual rates of drug overdose deaths for 2020–2022 were calculated using detailed multiple cause of death micro-data files from the National Vital Statistics System (NVSS). Drug overdose deaths were defined as records having an ICD10 code of X40-X44, X60-X64, X85, or Y10–14 as the underlying cause of death^25,26^. Counts of overdose deaths were then aggregated by municipality of residence, which was identified in the mortality records for U.S. Census places having populations of 100,000 or more. Rates of death per 100,000 population were computed using ACS 5-year municipal population estimates. For 209 cities with < 100,000 residents, municipal overdose rates were imputed according to spatial overlap with surrounding counties, using areal weighting with county overdose and county population estimates for 2020–2022.

### 2.4 Data Analysis

For descriptive analyses, municipal characteristics were categorized into quartiles based on the overall distribution across study cities, except for region, which was classified by subdivision rather than quartiles. Split by the presence or absence of a DNPO, counts of descriptive characteristics were determined within each quartile. A chi-squared test of independence was performed to test differences in the distribution of outcomes between municipalities with and without DNPOs. Of the 482 cities, 425 were used for descriptive analysis related to arrests due to missing arrest data within 57 cities. Of the cities excluded, 29 cities had DNPOs. R version 2025.09.2 + 418 was used for the analysis. The study protocol was reviewed and approved by the NYU Langone Health Institutional Review Board.

## 3. RESULTS

### 3.1 Descriptive Epidemiology

Of the 482 municipalities in our study, 202 (41.9%) had at least one DNPO. Among municipalities with any DNPO, most (n = 175, 86.6%) had one relevant law, 26 (12.9%) had two relevant laws, and one municipality (Long Beach, CA) had three relevant laws, for a total of 230 individual DNPOs across 202 municipalities. Table 1 presents the distribution of municipalities by nuisance activity category and activity type, and whether criminal penalties, eviction, or both were explicitly authorized. Of the 202 municipalities with at least one DNPO, 198 (41.1% of all cities; 98.0% of those with DNPO) had at least one law that explicitly defined drug activity as a nuisance, while the four remaining municipalities defined ‘any illegal activity’ as a nuisance. In addition, 87 of these municipalities (43.1%) also defined some ‘other nuisance’ (disorderly conduct, disturbing the peace, or non-drug related loitering) as a nuisance activity. 157 municipalities (77.7% of those with a DNPO) specifically identified drug or drug paraphernalia possession or use as a nuisance. Among municipalities with DNPOs, a total of 36.1% of municipalities authorized eviction as a method of abatement (14.4% with only eviction authorized and 21.8% with both eviction and criminal penalties authorized) and 55.5% of municipalities authorized criminal penalties (33.7% with only criminal penalties authorized and 21.8% with eviction and criminal penalties authorized) (Table 1).

Counts across nuisance categories were not mutually exclusive, as many municipalities designated multiple activities as nuisances. Combinations of nuisance categories across municipalities are summarized in Table 2. The most common configuration consisted solely of laws deeming drug activity as a nuisance (n = 65; 32.2% of all DNPOs). Other frequent combinations included: [1] drug activity and other nuisances (15.3%), [2] drug paraphernalia activity, drug activity, and other nuisances (14.9%) [3] drug paraphernalia activity and drug activity (13.4%).

**Figure 1:**
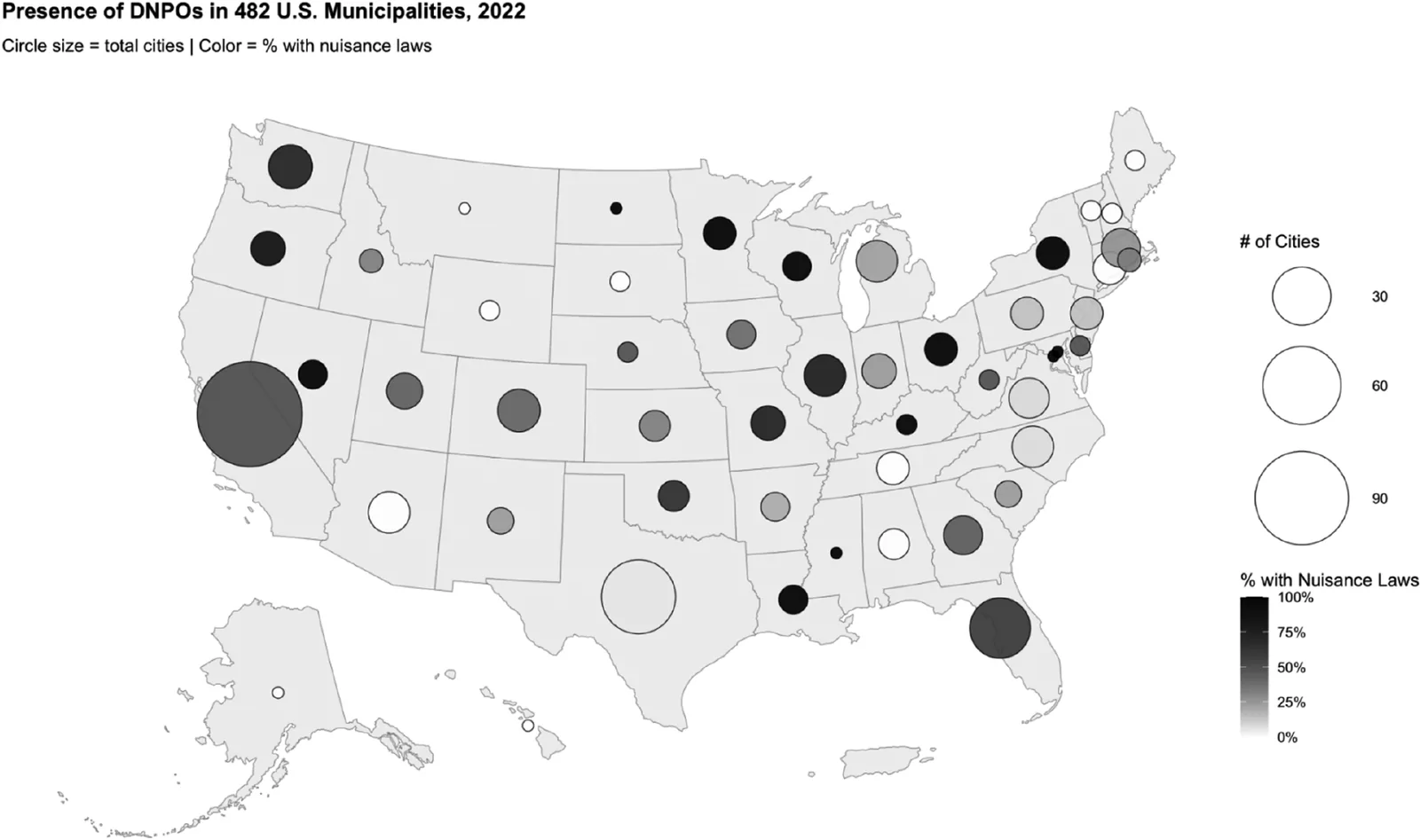
Distribution of presence of Drug-Related Nuisance Property Ordinances (DNPOs) across the United States. Presence of drug activity nuisance property ordinances within 482 municipalities across the 50 U.S. states and the District of Columbia. Circle size indicates the number of municipalities in the dataset within each state; shading indicates the percentage of municipalities within each state with DNPOs

Figure 1 illustrates the geographic distribution of municipalities with and without DNPOs, distinguishing between municipalities that authorize eviction as a method of abatement and those that do not. California (n = 117), Texas (n = 53), and Florida (n = 33) are the states with the largest representation of cities in the overall city sample (42.1%). California (n = 60; 29.7%), Florida (n = 20, 9.9%), and Washington (n = 10; 5.0%) are the states with the largest representation of cities in the subsect of cities with DNPOs (44.6% of all cities with DNPOs).

### 3.2 Drug-Related Nuisance Property Ordinance and Municipal-level Characteristics

Table 3 describes the distribution of demographic, socioeconomic, and crime-related variables for municipalities with DNPOs (n = 202). DNPOs were more likely to be found in municipalities with greater concentrations of Black, non-Hispanic residents (50.4% of cities in Quartile 4 (Q4) vs 31.4% of cities in Quartile 1(Q1)) or Other, non-Hispanic residents (47.1% in Q4 vs 29.8% in Q1), and having higher rates of poverty (44.6%−50.0% in Q2-Q4 vs 26.4% in Q1), unemployment (54.5% in Q4 vs 26.4% in Q1), renter-occupied households (47.1% in Q4 vs 28.9% in Q1), population density (49.6% in Q4 vs 30.6% in Q1), and lower median income (39.7%−51.7% in Q1-Q3 vs 27.3% in Q4). Cities in the lowest quartile of opioid and all-drug overdose rates had lower frequencies of nuisance laws, but the pattern was not linear. Regionally, the West North Central (61.3%), East North Central (58.7%), and Pacific (54.9%) regions had the highest frequency of cities with DNPOs, while New England (14.8%) and East South Central (18.8%) had the lowest. Arrest rates (drug offenses rates, property crime rates, and public order offense rates) also differed in distribution between municipalities with and without DNPOs (p < 0.05). Within the subset of municipalities with available arrest data, 173 cities possessed DNPOs. Cities with DNPOs were more likely to be in lower quartiles of drug offense arrests and higher quartiles of property crime and public order arrests.

## 4. DISCUSSION

Overall, nearly half of the 482 municipalities in our study had at least one DNPO. While drug-activity was the most common activity listed as a nuisance, other activities were often enumerated in tandem. Approximately 1 in 10 cities broadly defined any illegal activity as a nuisance, and nearly 1 in 5 classified disturbing the peace, disorderly conduct or loitering, activities with no clear parameters for violation, as nuisance activities. Over one-third of DNPOs specifically listed eviction as a method of abatement, and more than half of cities with DNPOs imposed some form of criminal penalty for violation. As the largest multi-municipal assessment of nuisance property ordinance statutory language and associated demographics to date, this national analysis extends prior smaller-scale research by demonstrating that nuisance laws, and the penalties attached to them, are widely adopted across U.S. municipalities and therefore, have the potential for broad population-level impact.

Our findings indicate that DNPOs are more commonly present in communities with greater socioeconomic and housing vulnerability. Municipalities with higher proportions of Black, non-Hispanic residents, lower median household income, higher poverty and unemployment, larger populations, and greater shares of renter-occupied housing were more likely to have a DNPO. Cities with the lowest overdose rates were less likely to have a DNPO than areas with moderate and higher rates of overdose. While these associations do not imply causality, they suggest that DNPOs are disproportionately concentrated in communities already facing structural disadvantage and heightened exposure to legal and housing-related risk. Race-based motivations for implementation of a nuisance property ordinance may violate state and federal law, including the Fair Housing Act’s prohibition against race discrimination^7,27–29^ These results are consistent with and extend prior literature,^3,7,13^ reinforcing the role of racial inequities in nuisance law adoption and highlighting additional previously unexamined municipal-level factors including economic indicators (lower median household income, higher poverty and unemployment) population size, geographic context, and housing composition associated with nuisance law presence.

Interestingly, lower drug arrest rates were associated with the presence of a DNPO. One possible explanation is that drug-related behaviors were addressed through nuisance enforcement mechanisms rather than traditional drug offense statutes in these municipalities. In contrast, municipalities with higher property crime arrest rates were more likely to be in cities with a nuisance law, findings consistent with the principle that nuisance laws penalize activities specifically on residential properties. Together, these findings highlight potential variability in how municipalities may utilize a nuisance property ordinance as third-party policing tactics.

With potential implications for housing stability, physical and mental health, and equity, the largest and clearest of such impacts is nuisance-triggered eviction. Kroeger et.al showed that in Ohio between 2000–2016, criminal activity nuisance ordinances increased eviction filing rates by about 16 percent and court ordered evictions by 14 percent^30^. Desmond et.al found that after a nuisance citation was triggered, 49% of property owners initiated formal eviction proceedings and 78% pursued informal eviction strategies^3^. Prior studies show that eviction risk is disproportionately borne by vulnerable populations. Across 39 states, although Black tenants comprised 19.9% of renters, they accounted for 32.7% of eviction filing defendants and, along with Hispanic renters, were more likely to face repeated filings at the same address^31^. Eviction filings are also more common in socioeconomically disadvantaged communities such as those with higher shares of single-mother households, youth populations, and limited job access, reinforcing cycles of housing insecurity among already marginalized groups^32^. Eviction filings against already vulnerable populations create a feedback loop that exacerbates poor housing outcomes.

Given over one-third of municipalities from our study authorized eviction as a legal method of abatement, these potential adverse effects may impact a broad range of populations across the United States. Eviction is associated with increased homelessness, job loss, psychological distress, and elevated mortality rates^8,11^. Among PWUD, eviction is linked to heightened overdose risk, syringe sharing, escalated drug use, and likelihood to remain unhoused^11,33–35^. Bradford et.al showed that, between 2003–2016, all counties in 46 states and the District of Columbia that showed higher eviction rates were associated with higher substance-related mortality rates^35^. In the same study, urban counties, over suburban and rural counties, were found to possess a strong net positive association between eviction and substance-related mortality^35^. Additionally, over 50% of municipalities from our study authorized criminal penalties for violation of nuisance property offenses, such as misdemeanor and felony convictions. These penalties pose further structural barriers to re-housing in settings where criminal records restrict access to market and non-market housing^36,37^.

This study has several limitations. Our sample was restricted to the most populous municipalities, which limits generalizability to smaller municipalities, rural municipalities, or unincorporated areas. Additionally, this analysis was limited to legal text and did not capture real-world implementation, enforcement practices, or interpretation by local officials. Accordingly, we are unable to assess how frequently DNPOs are applied within municipalities or whether the observed associations correspond to differential enforcement patterns across vulnerable communities. Finally, to streamline identification of nuisance property ordinances penalizing drug-related activity, we limited our search to six relevant categories [drug activities, drug paraphernalia activities, illegal activity disorderly conduct, disturbing the peace, and loitering]. However, municipalities may have employed alternative or broader language to capture drug-related behavior, which could have been missed in our search.

This study is the first to broadly demonstrate the importance of examining drug nuisance laws as an avenue to uncover systemic inequities. Given the disproportionate and harmful consequences of these laws, continued research is critical in understanding how these laws are enforced, maintained, and structured to penalize marginalized populations.

## Supplementary Material

Supplementary Files

This is a list of supplementary files associated with this preprint. Click to download.

- Table.1JoUH.docx
- Table.2JoUH.docx
- Table.3JoUH.docx

Tables 1 to 3 are available in the supplementary files section.
